# Identification of superconductivity in bilayer nickelate La_3_Ni_2_O_7_ under high pressure up to 100 GPa

**DOI:** 10.1093/nsr/nwaf220

**Published:** 2025-05-29

**Authors:** Jingyuan Li, Di Peng, Peiyue Ma, Hengyuan Zhang, Zhenfang Xing, Xing Huang, Chaoxin Huang, Mengwu Huo, Deyuan Hu, Zixian Dong, Xiang Chen, Tao Xie, Hongliang Dong, Hualei Sun, Qiaoshi Zeng, Ho-kwang Mao, Meng Wang

**Affiliations:** Center for Neutron Science and Technology, Guangdong Provincial Key Laboratory of Magnetoelectric Physics and Devices, School of Physics, Sun Yat-Sen University, Guangzhou 510275, China; Shanghai Key Laboratory of Material Frontiers Research in Extreme Environments (MFree), Institute for Shanghai Advanced Research in Physical Sciences (SHARPS), Shanghai 201203, China; Center for Neutron Science and Technology, Guangdong Provincial Key Laboratory of Magnetoelectric Physics and Devices, School of Physics, Sun Yat-Sen University, Guangzhou 510275, China; Center for Neutron Science and Technology, Guangdong Provincial Key Laboratory of Magnetoelectric Physics and Devices, School of Physics, Sun Yat-Sen University, Guangzhou 510275, China; Center for High Pressure Science & Technology Advanced Research, Shanghai 201203, China; Center for Neutron Science and Technology, Guangdong Provincial Key Laboratory of Magnetoelectric Physics and Devices, School of Physics, Sun Yat-Sen University, Guangzhou 510275, China; Center for Neutron Science and Technology, Guangdong Provincial Key Laboratory of Magnetoelectric Physics and Devices, School of Physics, Sun Yat-Sen University, Guangzhou 510275, China; Center for Neutron Science and Technology, Guangdong Provincial Key Laboratory of Magnetoelectric Physics and Devices, School of Physics, Sun Yat-Sen University, Guangzhou 510275, China; Center for Neutron Science and Technology, Guangdong Provincial Key Laboratory of Magnetoelectric Physics and Devices, School of Physics, Sun Yat-Sen University, Guangzhou 510275, China; Center for Neutron Science and Technology, Guangdong Provincial Key Laboratory of Magnetoelectric Physics and Devices, School of Physics, Sun Yat-Sen University, Guangzhou 510275, China; Center for Neutron Science and Technology, Guangdong Provincial Key Laboratory of Magnetoelectric Physics and Devices, School of Physics, Sun Yat-Sen University, Guangzhou 510275, China; Center for Neutron Science and Technology, Guangdong Provincial Key Laboratory of Magnetoelectric Physics and Devices, School of Physics, Sun Yat-Sen University, Guangzhou 510275, China; Shanghai Key Laboratory of Material Frontiers Research in Extreme Environments (MFree), Institute for Shanghai Advanced Research in Physical Sciences (SHARPS), Shanghai 201203, China; Center for High Pressure Science & Technology Advanced Research, Shanghai 201203, China; School of Science, Sun Yat-Sen University, Shenzhen 518107, China; Shanghai Key Laboratory of Material Frontiers Research in Extreme Environments (MFree), Institute for Shanghai Advanced Research in Physical Sciences (SHARPS), Shanghai 201203, China; Center for High Pressure Science & Technology Advanced Research, Shanghai 201203, China; Shanghai Key Laboratory of Material Frontiers Research in Extreme Environments (MFree), Institute for Shanghai Advanced Research in Physical Sciences (SHARPS), Shanghai 201203, China; Center for High Pressure Science & Technology Advanced Research, Shanghai 201203, China; Center for Neutron Science and Technology, Guangdong Provincial Key Laboratory of Magnetoelectric Physics and Devices, School of Physics, Sun Yat-Sen University, Guangzhou 510275, China

**Keywords:** nickelates, high-temperature superconductivity, Ruddlesden-Popper phase, high-pressure technique, Meissner effect

## Abstract

Identification of superconductivity in the Ruddlesden-Popper phases of nickelates under high pressure remains challenging. Here, we report a comprehensive study of the crystal structure, electrical resistance, and Meissner effect in single crystals of bilayer nickelate La_3_Ni_2_O_7_ under hydrostatic pressures up to 104 GPa. Using high-pressure X-ray diffraction, we observe a structural transition from an orthorhombic to a tetragonal phase above 40 GPa. Superconductivity emerges with a maximum onset transition temperature *T*_c_^onset^ of 83 K at 18.0 GPa, accompanied by zero resistance. The superconducting phase is gradually suppressed and vanishes above 80 GPa, forming a right-triangle-like superconducting region. Direct-current magnetic susceptibility measurements demonstrate the Meissner effect and reveal a superconducting volume fraction of ∼41% at 22.0 GPa and 20 K, confirming the bulk nature of superconductivity in La_3_Ni_2_O_7_. Our results highlight the intricate relationship between superconductivity, oxygen content, and structural transitions in this material.

## Teaser

The Meissner effect in La3Ni2O7 single crystals has been measured for the first time using a DC magnetic field in a SQUID, confirming bulk superconductivity. A comprehensive superconducting phase diagram is presented, extending up to 100 GPa.

## INTRODUCTION

The discovery of high-temperature superconductivity in cuprates has spurred extensive research into nickelates, which share similar lattice and electronic structures. In particular, the Ni^+^ ion in *Re*NiO_2_ (*Re *= La, Nd, Sm, etc.) exhibits a spin configuration analogous to Cu^2+^ in cuprates [1,2]. However, superconductivity in nickelates remained elusive until the discovery of a superconducting (SC) transition at *T*_c_ = 15 K in Nd_0.8_Sr_0.2_NiO_2_ thin films in 2019 [3–5]. Ni ions with a valence state close to Ni^+^ and a spin S = 1/2 in planar coordination with oxygen ions were thought to be crucial for the emergence of superconductivity in nickelates [2,6].

The recent observation of superconductivity in the Ruddlesden-Popper (RP) phase nickelate La_3_Ni_2_O_7_ with *T*_c_ ∼ 80 K has reignited interest in this field [7–12]. The SC phase in La_3_Ni_2_O_7_ is characterized by a bilayer structure with a ‘2222’ stacking sequence of NiO_6_ octahedra, which undergoes a structural transition from an orthorhombic *Amam* phase to a high-pressure orthorhombic *Fmmm* phase at 14.0 GPa [7]. This transition is accompanied by a change in the Ni-O-Ni bond angle along the *c*-axis from 168° to 180°. Scanning transmission electron microscopy (STEM) [13] and X-ray diffraction (XRD) studies [14] have confirmed the ‘2222’ stacking sequence in single crystals of La_3_Ni_2_O_7_ grown by the high-pressure floating zone method and polycrystalline samples of La_3_Ni_2_O_7_ [15] and La_2_PrNi_2_O_7_ [16] grown by the sol-gel method. Oxygen vacancies, particularly at the inner apical oxygen site shared by two NiO_6_ octahedra, have been visualized using STEM [13]. Further structural analysis under high pressure and low temperature has revealed a tetragonal *I*4*/mmm* phase associated with the SC state [17]. In addition, a new structure of La_3_Ni_2_O_7_ with an alternating monolayer-trilayer NiO_6_ octahedra stacking sequence, denoted as the ‘1313’ phase, was identified [18–21]. The properties of the distinct structure under high pressure remain unclear.

Despite these advances, several questions remain unresolved. For instance, the difficulty in achieving zero resistance and the weak suppression of *T*_c_ in La_3_Ni_2_O_7_ under pressures below 43.5 GPa have raised concerns about the nature of superconductivity in this material [7,9–11]. Additionally, the low SC volume fraction observed in alternating-current (AC) magnetic susceptibility measurements and the poor reproducibility of results have cast doubt on the bulk superconductivity in La_3_Ni_2_O_7_ [7,11,15,19]. It is essential to examine the nature of the superconductivity in the bilayer nickelate La_3_Ni_2_O_7_ and how this superconductivity evolves up to higher pressures.

In this work, we address these issues by conducting a detailed investigation of the high-pressure structural, electrical, and magnetic properties of La_3_Ni_2_O_7_. Using neon gas as a pressure-transmitting medium, we observe a structural transition from orthorhombic to tetragonal symmetry above 40 GPa. High-pressure transport measurements reveal a SC phase diagram with a maximum *T*_c_^onset^ of 83 K, which is suppressed above 80 GPa. Direct-current (DC) magnetic susceptibility measurements reveal the Meissner effect and confirm the bulk nature of superconductivity, with a SC volume fraction of ∼41% at 22.0 GPa. These findings provide new insights into the relationship between superconductivity, oxygen content, and structural transitions in La_3_Ni_2_O_7_.

## RESULTS

High-pressure synchrotron XRD measurements were performed at the BL15U1 beamline of the Shanghai Synchrotron Radiation Facility (SSRF) using a wavelength of λ = 0.6199 Å. Polycrystalline samples of La_3_Ni_2_O_7_ synthesized by the sol-gel method were used for these XRD measurements [15]. Single crystals of La_3_Ni_2_O_7_ from the same batch, as we investigated before, were used for diamond anvil cell (DAC) electrical transport and DC magnetic susceptibility measurements [7,14,22]. Both polycrystalline and single-crystal samples were confirmed to exhibit the bilayer RP phase structure (see Fig. S1). A custom-designed miniature DAC made of beryllium-copper alloy was employed for ultrasensitive DC magnetic susceptibility measurements, which were conducted using a magnetic property measurement system (MPMS, Quantum Design, UK). The identical setup has successfully measured the Meissner effect in La_4_Ni_3_O_10_ under pressure [23–26].

**Figure 1.**
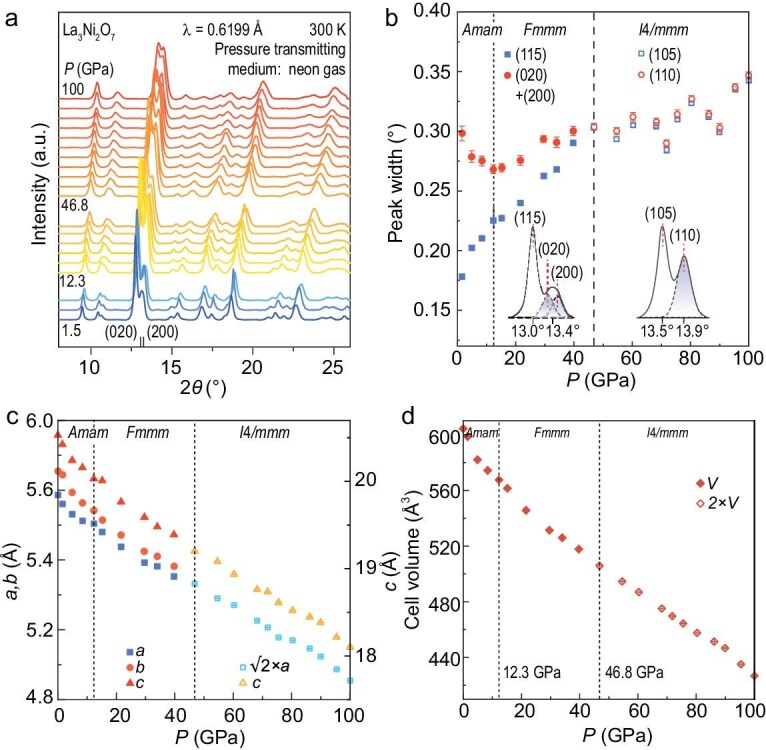
High-pressure structural characterizations of La_3_Ni_2_O_7_ up to 100 GPa. (a) Synchrotron XRD patterns of La_3_Ni_2_O_7_ under pressures from 1.5 to 100 GPa. The pressure transmitting medium (PTM) is neon gas. (b) Pressure dependence of the peak widths of (020)/(200) as a whole and (115) in the *Amam* space group. A merging of the peak widths at 12.3 GPa signals a structural transition from the *Amam* phase to the *Fmmm* phase. The peak widths of (020)/(200) become indistinguishable from those of (115) above 46.8 GPa, indicating a structural transition from *Fmmm* to tetragonal *I*4*/mmm* space group. The reflection indexes of (115) and (020)/(200) in the *Amam* space group change to (105) and (110) in the *I*4*/mmm* space group. The insets are the zoom-in experimental data. (c) Lattice parameters of La_3_Ni_2_O_7_ obtained from the synchrotron XRD data. (d) Cell volumes of La_3_Ni_2_O_7_.

Our previous synchrotron XRD results indicated a structural transition from the *Amam* to the *Fmmm* space group at ∼14 GPa when silicon oil was used as the pressure-transmitting medium (PTM) [7]. However, the pressure inhomogeneity induced by the solidification of silicon oil at 3 GPa and its glass-to-glass transitions at 10 and 16 GPa may have involved artifacts in the data analysis [27]. To mitigate these effects, we used neon gas as the PTM in the current high-pressure XRD study. An anomaly in the relative change of diffraction peaks was observed at 12.3 GPa (see Fig. S2), well above the crystallization pressure of 4.8 GPa and away from the non-hydrostatic pressure limit of 16.0 GPa for neon [28]. Figure 1b shows the pressure dependence of the peak widths of the (115) and (020)/(200) reflections. The peak widths broaden gradually with increasing pressure. The width of the (020)/(200) peak is broader than that of the (115) peak at low pressures due to the orthorhombicity, while they merge above 46.8 GPa, revealing a transition to a tetragonal structure (space group *I*4*/mmm*). A kink in the peak width of (020)/(200) at 12.3 GPa suggests a transition from the *Amam* to the *Fmmm* space group, as we proposed previously [7]. The refined lattice parameters are plotted in Fig. 1c and 1d.

**Figure 2.**
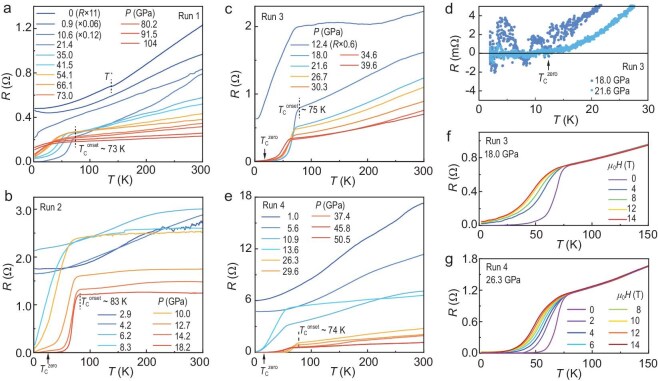
Temperature dependence of the in-plane resistance of La_3_Ni_2_O_7_ under various pressures. (a–c, e) High-pressure resistance curves from run 1 to run 4. The resistance of La_3_Ni_2_O_7_ from ambient pressure to 104 GPa is measured in run 1. A *T*_c_^onset^ ∼ 83 K is observed in run 2. (d) A zoom-in view of the resistance curves of run 3 under 18.0 and 21.6 GPa below 30 K. Zero resistance is achieved. (f, g) Field-dependent resistance curves at 18.0 GPa of run 3 and 26.3 GPa of run 4.

The superconductivity in La_3_Ni_2_O_7_ emerged from either a weakly insulating state or a metallic state at ambient pressure [7–11]. To ensure the reliability of our results, we performed high-pressure electrical transport measurements on five samples with similar dimensions (30 × 30 × 10 μm^3^) from the same batch. KBr was used as the PTM in these measurements. Figure 2a shows the resistance as a function of temperature at pressures ranging from 0 to 104 GPa. At ambient pressure, the resistance exhibits metallic behavior with an anomaly at T* ∼ 140 K, likely associated with a density-wave transition [14,26,29–32]. Under 0.9 GPa in run 1, the anomaly in resistance cannot be observed. A drop in resistance indicative of superconductivity is observed at 8 K and 10.6 GPa. The maximum *T*_c_^onset^ of 73 K is achieved at 21.4 GPa in run 1, with a residual resistance of 1 mΩ at 2 K. Superconductivity is gradually suppressed and vanishes above 80.2 GPa. Figures 2b–2e and S3 show the high-pressure transport measurements on the other samples. The maximum *T*_c_^onset^ reaches 83 K at 18.2 GPa in run 2, with zero resistance observed in runs 2, 3, and 4 (Fig. 2d), indicating high sample quality. The suppression of superconductivity by an external magnetic field is shown in Fig. 2f and 2g. A Ginzberg-Landau fitting yields an upper critical field of 126 T for run 4.

**Figure 3.**
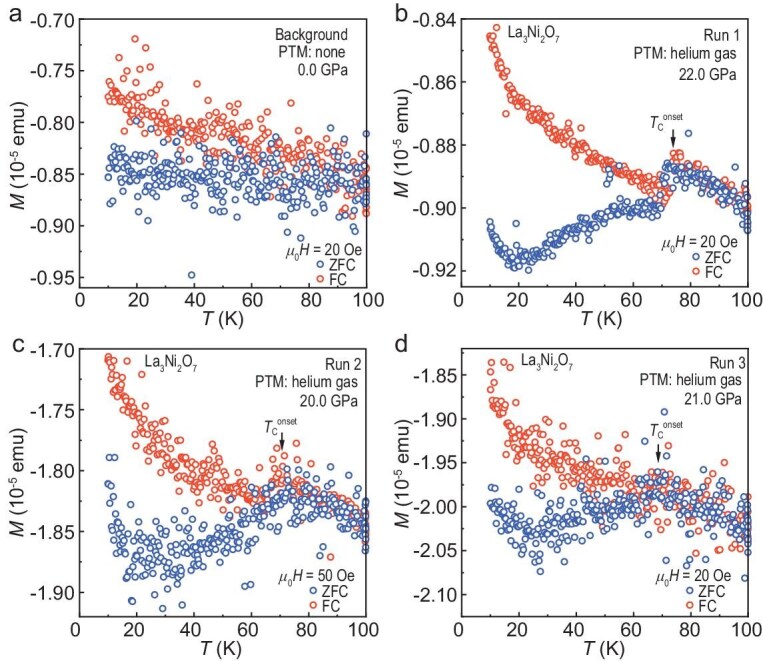
Direct-current magnetic susceptibility measurements of La_3_Ni_2_O_7_ under pressure. Zero-field cooling (ZFC) and field cooling (FC) curves are measured with a field perpendicular to the *ab* plane. (a) Background signals of the high-pressure cell without a sample at ambient pressure. (b–d) Evident Meissner effect induced diamagnetic signals of pressurized La_3_Ni_2_O_7_ single crystals from run 1 to run 3. Both ZFC and FC curves show prominent diamagnetic responses. The black arrows indicate the onset temperatures of superconductivity.

To further investigate the nature of the superconductivity in La_3_Ni_2_O_7_ under pressure, we performed high-pressure DC magnetic susceptibility measurements to detect the Meissner effect. The DAC used in this experiment featured a pair of diamond anvils, each with a diameter of 400 μm. Non-magnetic rhenium gaskets were adopted to minimize the interference of the magnetic measurements. In run 1 of the DC susceptibility measurements, a single crystal of La_3_Ni_2_O_7_ with dimensions of ∼180 μm in diameter and 20 μm in thickness was placed in the sample chamber. Helium gas was used as the PTM to ensure optimal hydrostatic conditions. Distinct from the nearly constant background signals in Fig. 3a, significant diamagnetic behavior was observed around *T*_c_^onset^ ∼ 76 K under 22.0 GPa in both zero-field cooling (ZFC) and field cooling (FC) measurements in run 1 (Fig. 3b). The SC volume fraction was estimated to be 14% and 12% at 67 K for the ZFC and FC measurements, respectively. At lower temperatures, where the background susceptibility is unknown, the FC data were treated as the background and subtracted from the ZFC data to determine the SC volume fraction. This procedure yielded 41%, 31%, and 33% for runs 1, 2, and 3, respectively, as illustrated in Fig. 3b–3d. We note that the choice of background will lead to an underestimation of the SC volume fraction. Further details on the estimation method are provided in the Supplementary material. These results prove the Meissner effect and confirm the bulk superconductivity in La_3_Ni_2_O_7_ single crystals.

## DISCUSSION

**Figure 4.**
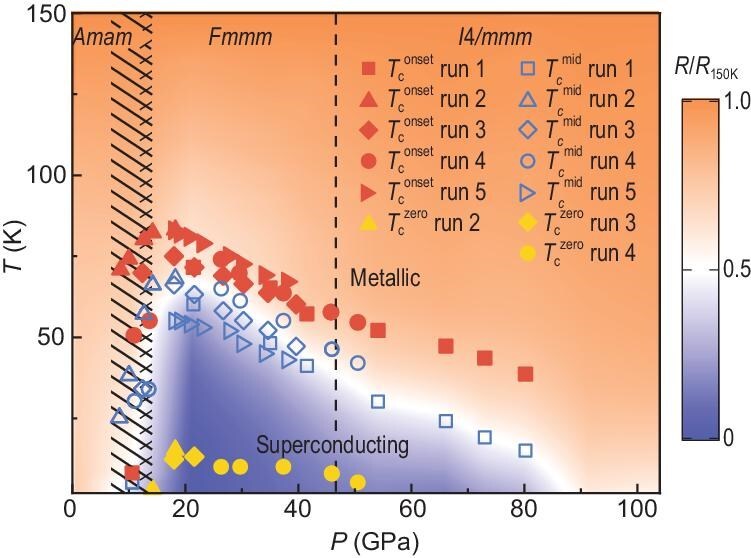
The superconducting phase diagram of La_3_Ni_2_O_7_ single crystals under ambient pressure to 104 GPa. The red solid symbols represent the onset temperatures of superconductivity *T*_c_^onset^ obtained from five runs. The blue hollow symbols represent the middle temperatures *T*_c_^mid^ of the superconducting transition defined by the temperature corresponding to the resistance of *R*_mid_ =  (*R*_onset _+ *R*_2K_)/2. The yellow dots mark the zero resistance temperatures *T*_c_^zero^ of run 2 to run 4. The color of the ground shows the data of run 1. Structural transition pressures are indicated by the black striped lines and the dashed line.

The phase diagram and structural transitions of La_3_Ni_2_O_7_ under high pressure are summarized in Fig. 4. The onset transition temperature *T*_c_^onset^ is defined as the temperature at which the resistance drops, while the *T*_c_^mid^ is the temperature at which the resistance is in the middle between that at the *T*_c_^onset^ and 2 K. The background color scale represents the relative resistance change normalized to the value at 150 K in run 1. Superconductivity emerges as roughly coincidental with the structural transition from *Amam* to *Fmmm*, likely due to Fermi surface reconstruction or enhanced interlayer magnetic exchange coupling [33–44]. The *T*_c_^onset^ reaches a maximum of 83 K at 18.0 GPa and decreases with increasing pressure, forming a right-triangle-like SC region. The *T*_c_^onset^ drops to 38 K at 80.2 GPa, indicating a robust bulk SC phase in La_3_Ni_2_O_7_. The orthorhombic structure revealed by XRD between 12.3 and 46.8 GPa suggests a tetragonal structure is not essential for the pressure-induced superconductivity in La_3_Ni_2_O_7_.

The emergence of superconductivity in La_3_Ni_2_O_7_ under high pressure is highly dependent on sample quality and pressure homogeneity [7–9,11]. Compared to La_2_NiO_4_ and La_4_Ni_3_O_10_, the bilayer La_3_Ni_2_O_7_ is a metastable phase with a narrower oxygen pressure window of 10–18 bar during the single crystal growth [45]. This complicates the sample synthesis, and sample inhomogeneity is hard to avoid. The other intergrowth phases, such as La_2_NiO_4_, La_4_Ni_3_O_10_, and some other stacking sequences, are also possible [18,21,46]. While zero resistance and the Meissner effect have been observed in high quality single crystals, zero resistance is only achieved in small samples with a typical size of 30 × 30 × 10 μm^3^ in our measurements. The SC volume fraction varies from a few percent in previous studies to ∼41% in this work, likely due to variation in oxygen vacancy. STEM measurements have revealed that oxygen vacancies, particularly at the inner apical oxygen site, play a crucial role in suppressing superconductivity [13]. The inner apical oxygen is directly involved in the superexchange magnetic interactions between nickel ions along the *c*-axis and affects the splitting of the bonding and antibonding states of the 3d_z^2^_ orbitals [7,39,47–50]. According to theoretical analysis, interlayer coupling plays an important role in the superconductivity of pressurized La_3_Ni_2_O_7_ [33,34,36,42–44,51–54]. Inelastic neutron scattering [55] and resonance inelastic X-ray scattering measurements [31] indeed reveal a strong interlayer coupling in La_3_Ni_2_O_7_ compared to the dominant intralayer couplings in copper-based [56] and iron-based superconductors [57]. It is reasonable to argue that the inner apical oxygen vacancies will suppress superconductivity in the bilayer nickelate under pressure.

## CONCLUSION

In conclusion, we have conducted a comprehensive study of the structural and SC properties of La_3_Ni_2_O_7_ under high pressure. Our results reveal a structural transition from orthorhombic to tetragonal symmetry above 46.8 GPa and a right-triangle-like SC phase diagram with a maximum *T*_c_^onset^ of 83 K. The SC phase is suppressed above 80 GPa, and DC magnetic susceptibility measurements reveal the Meissner effect and confirm the bulk nature of superconductivity with a SC volume fraction of ∼41% at 22.0 GPa and 20 K. These findings provide new insights into the relationship between superconductivity, oxygen content, and structural transitions in La_3_Ni_2_O_7_, paving the way for further exploration of high-temperature superconductivity in nickelates.

## Supplementary Material

nwaf220_Supplemental_File
