# Supplementary material for: Identification of superconductivity in bilayer nickelate La3Ni2O7 under high pressure up to 100 GPa
Source: Natl Sci Rev. 2025 May 29;12(10):nwaf220. doi: 10.1093/nsr/nwaf220 (PMC12485610; doi:10.1093/nsr/nwaf220)
Supplement: nwaf220_Supplemental_File [file nwaf220_supplemental_file.pdf]

Supplementary Information: Identification of Superconductivity in Bilayer Nickelate $La_3Ni_2O_7$ under High Pressure up to 100 GPa

Jingyuan Li[1,†], Di Peng[2,†], Peiyue Ma[1], Hengyuan Zhang[1], Zhenfang Xing[3], Xing Huang[1], Chaoxin Huang[1], Mengwu Huo[1], Deyuan Hu[1], Zixian Dong[1], Xiang Chen[1], Tao Xie[1], Hongliang Dong[2,3], Hualei Sun[4,*], Qiaoshi Zeng[2,3,*], Ho-kwang Mao[2,3], Meng Wang[1,*]

[1]*Center for Neutron Science and Technology, Guangdong Provincial Key Laboratory of Magnetoelectric Physics and Devices, School of Physics, Sun Yat-Sen University, Guangzhou, Guangdong 510275, China*

[2]*Shanghai Key Laboratory of Material Frontiers Research in Extreme Environments (MFree), Institute for Shanghai Advanced Research in Physical Sciences (SHARPS), Shanghai 201203, China*

[3]*Center for High Pressure Science & Technology Advanced Research, Shanghai, 201203, China*

[4]*School of Science, Sun Yat-Sen University, Shenzhen, Guangdong 518107, China*

[†]These authors contributed equally to this work.

[*]E-mail: sunhlei@mail.sysu.edu.cn, zengqs@hpstar.ac.cn, wangmeng5@mail.sysu.edu.cn

Sample information

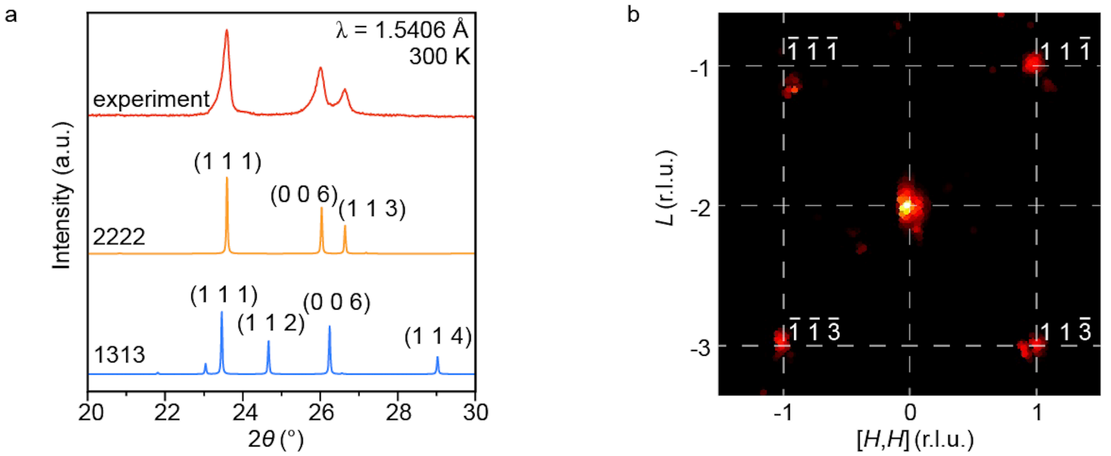

FIG. S1. Structural characterizations of $La_3Ni_2O_7$ at ambient pressure. (a) Powder X-ray diffraction (XRD) pattern at ambient pressure (red line). The simulated XRD patterns of the 2222-phase (orange line) and 1313-phase (blue line) are compared below. (b) A slice of a single-crystal XRD pattern in the (HH*L*) plane. The (1 1 3) peak is present while the (1 1 2) peak is absent, consistent with the '2222' bilayer structure.

High-pressure XRD analysis

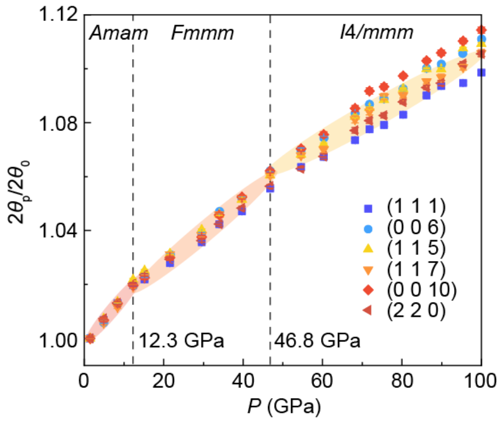

FIG. S2. Pressure dependence of the peak positions of six isolated Bragg peaks. The peaks are labeled by the Miller indices in the *Amam* space group.

TABLE S1 Crystal parameters of pressurized $La_3Ni_2O_7$ obtained from the Rietveld refinements.

Pressure (GPa)	*a* (Å)	*b* (Å)	*c* (Å)	Space group	R_{wp} (%)	R_p (%)	GOF
0	5.3964(2)	5.4549(1)	20.5293(8)	*Amam*	4.30	6.50	2.51
1.5	5.3605(8)	5.4448(8)	20.424(4)	*Amam*	6.70	9.40	0.86
4.9	5.3309(7)	5.3939(7)	20.242(3)	*Amam*	9.31	10.52	0.90
8.4	5.3123(6)	5.3631(7)	20.159(3)	*Amam*	8.63	10.10	0.89
12.3	5.3041(9)	5.3417(8)	20.031(4)	*Fmmm*	10.47	13.80	1.20
15.2	5.2806(8)	5.3144(9)	20.010(4)	*Fmmm*	10.02	12.84	1.14
21.6	5.2376(7)	5.2714(8)	19.766(2)	*Fmmm*	9.60	12.85	1.18
29.6	5.1925(9)	5.2248(8)	19.590(3)	*Fmmm*	10.32	11.57	1.15
34.0	5.1820(7)	5.2108(5)	19.480(2)	*Fmmm*	7.73	9.19	0.99
39.7	5.1531(8)	5.1815(6)	19.390(3)	*Fmmm*	7.45	8.06	0.77
46.8	3.6296(5)	3.6296(5)	19.201(5)	*I4/mmm*	8.64	9.46	0.84
54.6	3.6001(6)	3.6001(6)	19.083(3)	*I4/mmm*	9.21	10.50	0.97
60.3	3.5858(6)	3.5858(6)	18.935(4)	*I4/mmm*	9.10	11.60	0.91
68.2	3.5547(6)	3.5547(6)	18.761(4)	*I4/mmm*	8.76	10.80	0.71
71.8	3.5408(8)	3.5408(8)	18.734(5)	*I4/mmm*	10.90	13.20	1.04
75.5	3.5206(7)	3.5206(7)	18.613(4)	*I4/mmm*	11.60	14.60	1.22
80.3	3.5148(6)	3.5148(6)	18.522(4)	*I4/mmm*	10.00	12.00	0.96
86.2	3.4980(7)	3.4980(7)	18.447(4)	*I4/mmm*	11.20	15.90	1.65
89.9	3.4822(9)	3.4822(9)	18.384(5)	*I4/mmm*	12.20	14.90	1.45
95.4	3.4560(8)	3.4560(8)	18.216(5)	*I4/mmm*	11.90	15.20	0.98
100.0	3.433(1)	3.433(1)	18.101(6)	*I4/mmm*	12.60	14.50	1.56

High-pressure transport measurement

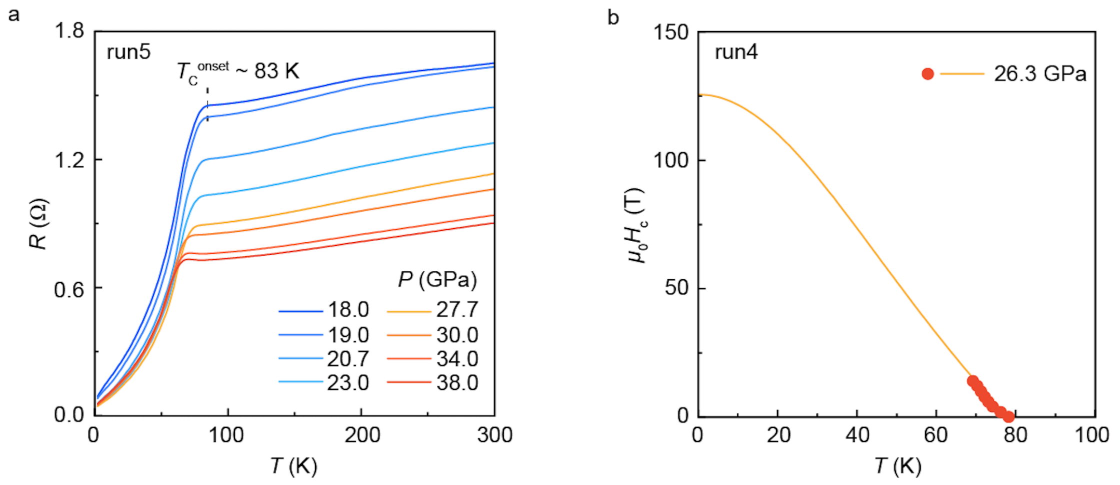

FIG. S3. (a) High-pressure resistance curves of run 5. (b) The Ginzberg-Landau fitting of the upper critical field $\mu_0 H_{c2}$ of run 4 under 26.3 GPa.

Superconducting volume fraction analysis

The measured susceptibility value in the international system of units is converted from the Gaussian units by

$$\chi_{0-sI} = 4\pi\chi_{CGS} = \frac{4\pi M}{HV}$$

where M, H, and V represent the magnetization of the sample, the external field, and the sample volume. The difference in magnetization between FC and ZFC (ΔM) at 20 K was adopted to calculate the SC volume fraction. A background subtraction process is needed to extract the diamagnetic signal induced by the sample. Generally, the magnetic susceptibility signal of the empty cell (with gasket but without loading the sample) is used as the background. However, after loading the sample, the background signal usually changes with increasing pressure. We adopt the FC curves as the background to avoid the inaccuracy of using the empty cell background. In principle, the factual ZFC background in the superconducting state should lie above the FC curve; this method would underestimate the magnitude of the diamagnetic susceptibility of the sample. After subtracting the FC susceptibility as background, the diamagnetization ΔM is -5.08×10^{-7} emu at 20 K under 22 GPa in run 1. The external field H is 20 Oe. The sample used in run 1 is approximately a cylinder with d = 180 μm in diameter and h = 20 μm in thickness. The value of χ_{0-sI} of run 1 at 20 K is -0.627.

The effect of geometric demagnetization was considered and deducted. The demagnetizing factor N of the sample can be calculated by[1]

$$N^{-1} \approx 1 + 1.6\frac{h}{d}$$

The demagnetizing factor of sample in run1 yields N = 0.849, and this value barely changes under pressure because the lattice contraction remains proportional under hydrostatic pressure. Finally, the adjusted susceptibility χ_{sI} of sample is calculated based on

$$N = \frac{1}{\chi_{0-sI}} - \frac{1}{\chi_{sI}}$$

Using the measured susceptibility χ_{0-sI} and the demagnetizing factor N, we obtain the adjusted susceptibility of run 1 at 22 GPa is χ_{sI} = -0.409 at 20 K. This result means a superconducting volume fraction of 40.9% is achieved in $La_3Ni_2O_7$. The susceptibility measurements for run 2 and run 3 yield χ_{sI} = -0.310 and -0.332 at 20 K, indicating superconducting volume fraction of 31.0% and 33.2%, respectively.

TABLE S2 Details of superconducting volume fraction calculation at 20 K.

run	ΔM (emu)	*d* (μm)	*h* (μm)	*V* ($\times10^{-7}$cm^3)	*N*	*H* (Oe)	χ_{0-sI}	χ_{sI}
**1**	-5.080×10^{-7}	180	20	5.089	0.849	20	-0.627	-0.409
**2**	-1.059×10^{-6}	200	20	6.283	0.862	50	-0.424	-0.310
**3**	-8.620×10^{-7}	270	20	11.45	0.894	20	-0.473	-0.332

References

1. Prozorov R and Kogan VG. *Phys. Rev. Appl.* 2018; **10**: 14030.
